# *VAMP2* Expression and Genotype Are Possible Discriminators in Different Forms of Dementia

**DOI:** 10.3389/fnagi.2022.858162

**Published:** 2022-03-14

**Authors:** Andrea Saul Costa, Evelyn Ferri, Franca Rosa Guerini, Paolo Dionigi Rossi, Beatrice Arosio, Mario Clerici

**Affiliations:** ^1^IRCCS Fondazione Don Carlo Gnocchi ONLUS, Milan, Italy; ^2^Geriatric Unit, Fondazione IRCCS Ca’ Granda Ospedale Maggiore Policlinico, Milan, Italy; ^3^Department of Clinical Sciences and Community Health, University of Milan, Milan, Italy; ^4^Department of Pathophysiology and Transplantation, University of Milan, Milan, Italy

**Keywords:** dementia, mixed dementia, SNARE complex, *VAMP2*, gene expression, biomarker

## Abstract

Vascular alterations often overlap with neurodegeneration, resulting in mixed forms of dementia (MD) that are hard to differentiate from Alzheimer’s Disease (AD). The 26 bp intergenic polymorphism of *VAMP2*, a key component of SNARE complex, as well as its mRNA and protein levels are associated with neurological diseases. We evaluated *ApoE4* and *VAMP2* 26 bp Ins/Del genotype distribution in 177 AD, 132 MD, 115 Mild Cognitive Impairment (MCI) and 250 individuals without cognitive decline (CT), as well as *VAMP2* gene expression in a subset of 73 AD, 122 MD, 103 MCI and 140 CT. Forty-two MCI evolved to AD (22 MCI-AD) or MD (20 MCI-MD) over time. *VAMP2* mRNA was higher in MD compared to AD (*p* = 0.0013) and CT (*p* = 0.0017), and in MCI-MD compared to MCI-AD (*p* < 0.001) after correcting for age, gender, MMSE and *ApoE4* +/− covariates (*p*_*c*_ = 0.004). A higher *VAMP2* expression was observed in subjects carrying the minor allele Del compared to those carrying the Ins/Ins genotype (*p* = 0.012). Finally, Del/Del genotype was more frequently carried by MD/MCI-MD compared to CT (*p*_*c*_ = 0.036). These results suggest that *VAMP2* mRNA expression can discriminate mixed form of dementia from AD, possibly being a biomarker of AD evolution in MCI patients.

## Introduction

Dementia is a common condition of the elderly characterized by multiple cognitive impairments leading to high disability. This clinical condition results from heterogeneous neurodegenerative and/or cerebrovascular diseases characterized by selective neuronal loss and by intra- or extracellular deposition of proteins (Prusiner, 2001) and/or by blood vessel blockage leading to dead tissue or bleeding area in the brain (Alzheimer’s Association, 2021). Neuropathological classification depends on the anatomical distribution of neuronal loss and cellular distribution of specific aggregated proteins (Dickson, 2005).

Between the cognitive changes of normal aging and those associated with dementia there is an intermediate stage called Mild Cognitive Impairment (MCI), which is characterized by memory deficits that do not impair global cognitive functions or daily lifestyle and activities (Forlenza et al., 2013). Neuropathological studies showed that synaptic loss is already evident in MCI (Counts et al., 2014) and MCI subjects exhibit loss of pre- and postsynaptic proteins (Pham et al., 2010). Unfortunately, MCI is often the initial step toward development of different kinds of dementia, including Alzheimer’s Disease (AD), Vascular dementia (VAD), Dementia with Lewy Body (DLB) and Frontotemporal Dementia (FTD) (Alzheimer’s Association, 2021).

In older people vascular alteration often overlap with neurodegenerative pathology (Schneider and Bennett, 2010; Attems and Jellinger, 2014), making the diagnosis process hard. For this reason, it would be of great importance to find out biomarkers able to predict how MCI condition can develop on the different kind of dementias. The release of neurotransmitters in the synaptic cleft, through the fusion of synaptic vesicles with the presynaptic membrane, is the basis of neuronal communication (Kavalali, 2015). To this regard the *N*-ethylmaleimide-sensitive factor attachment protein receptor (SNARE) complex, a fusion machinery that provides the energy required for the synaptic vesicles fusion with the presynaptic membrane, plays a crucial role (Weber et al., 1998) and defects in this machinery are associated with a range of neurological disorders associated to dementia (Guerini et al., 2014; Costa et al., 2019; Melland et al., 2021). The SNARE complex includes three specific proteins: the Syntaxin1a (STX1a), the synaptosomal-associated protein of 25 kDa (SNAP-25) and the vesicle associated membrane protein 2 (VAMP2). VAMP2 plays a fundamental role in the SNARE complex formation and functionality. The *VAMP2* gene is located on chromosome 17p13.1 and consists of 6 exons that encode for the SNARE complex key protein required for membrane fusion. A *VAMP2* gene 26 bp Ins/Del polymorphism, located in a intergenic region, suspected to be involved in gene expression, distant 2 kb from the 3′ flanking region of *VAMP2* was associated with neurological disease like Attention-deficit hyperactivity disorder and with neurodegenerative disease like Multiple Sclerosis (Sánchez-Mora et al., 2013; Kenar et al., 2014; Yalın et al., 2019). Notably, a reduction of *VAMP2* expression in the hippocampus and the entorhinal cortex was found to be associated with cognitive decline (Sze et al., 2000) and AD brains have a reduced level of VAMP2, STX1a and SNAP-25 compared to control brains (Pham et al., 2010).

Considering the crucial role of VAMP2 in the SNARE complex assembly, we evaluated whether VAMP2 could be a novel peripheral biomarker that can distinguish among different forms of cognitive impairment in old persons. For this purpose, *VAMP2* gene variant 26 bp Ins/Del and *VAMP2* mRNA expression in human peripheral blood mononuclear cells (PBMCs) were analyzed in patients with a diagnosis of either MCI, AD, or mixed dementia (MD).

## Materials and Methods

### Patients and Controls

The cohort consisted of 674 community-dwelling adults aged 63–100 years enrolled at Geriatric Unit of the Fondazione IRCCS Ca’ Granda Ospedale Maggiore Policlinico, Milan, Italy. All participants were admitted to the Geriatric Unit for the investigation of a suspect cognitive decline and their data recorded in Registro di Raccolta Dati della Unità di Geriatria (REGE). One hundred and seventy-seven individuals suffered from AD and 132 from MD. One hundred and fifteen had a diagnosis of MCI; 42 out of them evolved in either AD (*N* = 22: MCI-AD) or MD (*N* = 20: MCI-MD) over 2 years of the study period. Two hundred and fifty sex-matched subjects without cognitive decline (CT) were enrolled in the study as well.

Information about medical history, physical examination, and neurocognitive assessment (i.e., Mini Mental State Examination-MMSE, Clock Drawing Test-CDT), neuropsychological tests-NPS (i.e., Trail Making Test, verbal fluency test, digit span forward and backward tests, verbal learning tests, Token Test, Rey’s figure copy and delayed recall, Raven Colored Progressive Matrices), were recorded for all participants. The diagnosis of AD was made according to the criteria by Dubois et al. (2014), while MCI patients met the criteria outlined by Petersen (2004). Individuals showing the presence of both neurodegenerative and vascular findings were diagnosed as MD (Iadecola, 2010). CT were subjects with an MMSE score ≥ 27, the neuropsychological (NPS) tests negative for dementia and no neurological or psychiatric disorders.

The study was conducted in accordance with the declaration of Helsinki and the research protocol was approved by the Ethical Committee of the IRCCS Fondazione Ca’ Granda Ospedale Maggiore Policlinico (protocol number: 00223248). Informed consent to participate in the study has been signed by all participants.

### *VAMP2* and *ApoE* Genotyping

Genomic DNA was extracted using a previously described salting-out method (Miller et al., 1988) and its concentration and purity were determined by the means of spectrophotometric analysis. The *VAMP2* gene 26 bp Ins/Del polymorphism was genotyped by polymerase chain reaction (PCR) with a VeritiPro Thermal Cycler (Applied Biosystems by Life Technologies, Foster City, CA, United States), using *VAMP2* F-5′-ACAAAGTGCGCCTTATACGC-3′ and *VAMP2* R-5′-GATTTTCCTTGACGACACTC-3′ primers as previously described (Falbo et al., 2002). Amplicons (10 μL) were detected by electrophoresis on a 3% agarose gel. Finally, the *ApoE* genotype was determined as previously described (Ferri et al., 2019). In brief, a 244 bp *ApoE* fragment was amplified by PCR (step 1: 96 °C for 5 min; step 2 for 30 cycles: 95 °C for 1 min, 60 °C for 1.10 min, and 70 °C for 2 min; step 3: 70 °C for 10 min) with the primer pairs: 5′-GATCAAGCTTCCAATCACAGGCAGGAAG-3′ and 5′-GATCCGGCCGCACACGTCCTCCATG-3′. The amplified fragment was digested by using the *Hha*I enzyme and the products were visualized on agarose gel.

### *VAMP2* Gene Expression

Peripheral blood mononuclear cells (PBMCs) were isolated by density gradient (Lympholyte-H, Cedarlane, Canada). Total RNA was extracted from PBMCs using the Chomczynski and Sacchi’s modified method (Chomczynski and Sacchi, 2006). Two micrograms of total RNA were reverse-transcribed using the SuperScript VILOTM cDNA Synthesis Kit (Invitrogen by Thermo Fisher Scientific, Massachusetts, United States). Quantitative PCRs were performed in the OpenArray^®^ system QuantStudio 12K Flex Real-Time PCR System (Applied Biosystems by Thermo Fisher Scientific, Massachusetts, United States). For the *VAMP2* gene expression analysis, qPCR reactions based on TaqMan probes Hs00360269_m1 (Thermo Fisher Scientific) were performed using high-performance OpenArray^®^ chip. Three genes have been selected as endogenous according to their stable expression in human cells (*GAPDH*, *ACTB* and *18S*) and included into the OpenArray^®^ chip. One hundred and twenty ng of every cDNA sample (1.2 μL of each) were mixed with 1.3 μL of PCR-grade water and 2.5 μL of TaqMan™ OpenArray^®^ Real-Time PCR Master Mix (Applied Biosystems by Thermo Fisher Scientific, Massachusetts, United States). Samples were loaded in duplicate into Open-Array^®^ plates. For gene expression analysis, Ct values were obtained using the Thermo Fisher ConnectTM (Thermofisher) online application, and the Relative Quantification (RQ) software.

### Statistical Analysis

Pearson’s chi-square test was performed to compare categorical variables, to exclude any deviation of SNP genotype distribution from Hardy–Weinberg equilibrium and to evaluate case-control differences of SNP distribution. The distribution of demographic and clinical parameters was evaluated by Kolmogorov-Smirnov test to assess possible deviations from the Gaussian model; parametric data were reported as mean ± standard deviation, whereas non-parametric data were shown as median and interquartile range (IQR: 25th–75th percentile). Regarding the continuous values, parametric data were analyzed using the Analysis of Variance (ANOVA) and Student’s *t*-test, whereas non-parametric data were analyzed using the Kruskal–Wallis test and the Mann–Whitney test. A binomial logistic regression model (with forward stepwise selection) was computed, considering pathology status as response variable (MCI-AD vs. MCI-MD) and *VAMP2* expression as explanatory variable and inserting age, gender, MMSE and *ApoE4* positivity as covariates. *p*-values corresponding to < 0.05 were described as statistically significant in the text. Corrected *p*-values (p_*c*_) are reported for statistical analysis corrected for covariates. Data of *VAMP2* mRNA expression was used in Receiver operating characteristic (ROC) analysis to draw the ROC curve and to calculate the area under the curve (AUC). Statistical analyses were accomplished using commercial software (IBM SPSS Statistic 26.0, IBM Inc., Chicago, IL, United States).

## Results

### Characteristics of the Cohort

Demographic and clinical characteristics of the cohort are summarized in Table 1. Sex distribution was similar in all the groups examined. Age was different among the four groups (*p* < 0.001) with CT and MD subjects being older than MCI and AD patients (*p* = 0.03 and *p* < 0.001 for both, respectively). As expected, the MMSE score was different among the four groups (*p* < 0.001) with AD and MD patients having a significant lower MMSE score compared to CT and MCI (*p* < 0.001 for all the comparisons), as well as for the MCI compared to CT (*p* < 0.001). The distribution of the *ApoE4* variant showed a significant difference among the four groups (*p* < 0.001), with the *ApoE4* allele being more frequent in AD compared to CT, MCI and MD patients (AD vs. CT: *p* < 0.001; OR: 3.76; 95% CI: 2.43–5.89; AD vs. MCI: *p* < 0.001; OR: 2.57; 95% CI: 1.54–4.33; AD vs. MD: *p* < 0.001; OR: 2.20; 95% CI: 1.34–3.64). Similarly, the *ApoE4* variant was significantly more frequent in MD patients compared to CT (*p* = 0.043; OR: 1.71; 95% CI: 1.02–2.86). Forty-two of the 115 MCI with known clinical evolution in dementia were categorized according to disease progression: 22 evolved to AD (MCI-AD) and 20 to MD (MCI-MD). Demographic and clinical characteristics of these two sub-groups are summarized in Table 2. Sex distribution, MMSE and *ApoE4* distribution were comparable between the groups. MCI-MD were older than the MCI-AD patients (*p* = 0.021) (Table 2).

**TABLE 1:** Demographic and clinical characteristics of the cohort.

	CT	AD patients	MD patients	MCI	*p*-values
n.	250	177	132	115	
Gender (M:F)	82:168	52:125	40:92	45:70	*p* = 0.33
Age, y	79.35 ± 5.93^1^	77.54 ± 5.07	80.34 ± 4.86^2^	77.94 ± 5.42	***p* < 0.001**
MMSE	29.0; 27.0–30.0	21.0; 18.3–23.7^3^	21.2; 18.0–24.0^3^	27.0; 26.0–28.0^4^	***p* < 0.001**
*ApoE* ε4 carriers (%)	20.4	49.1^5^	30.4^6^	27.3	***p* < 0.001**

*CT, subject without cognitive decline; AD, subjects with Alzheimer’s disease; MD, subjects with mixed Dementia; MCI, subjects with Mild Cognitive Impairment; MMSE, Mini-Mental State Examination.*

*^1^p < 0.03 vs. MCI and vs. AD; ^2^p < 0.001 vs. MCI and vs. AD; ^3^p < 0.001 for AD vs. CT; AD vs. MCI; MD vs. CT; MD vs. MCI; ^4^p < 0.003 vs. CT; ^5^p < 0.001 vs. CT, vs. MCI and vs. MD; ^6^p = 0.043 vs. CT. Bold values are indicate a statistically significant p-value.*

**TABLE 2:** Demographic and clinical characteristics of the MCI evolved in dementia.

	MCI-AD	MCI-MD	*p*-values
n.	22	20	
Gender (M:F)	8:14	11:9	*p* = 0.24
Age, y	76.45 ± 5.45	80.01 ± 3.95	***p* = 0.021**
MMSE	26.0; 25.0–27.0	26.0; 24.0–27.7	*p* = 0.52
*ApoE* ε4 carriers (%)	50.0	40.0	*p* = 0.53

*MCI-AD, Mild Cognitive Impairment evolved to Alzheimer’s Disease; MCI-MD, Mild Cognitive Impairment evolved to Mixed Dementia; MMSE, Mini Mental State Examination. Bold values are indicate a statistically significant p-value.*

### *VAMP2* mRNA Expression According to *VAMP2* 26 bp Ins/Del Polymorphism

The *VAMP2* mRNA expression level was evaluated according to the *VAMP2* 26 bp Ins/Del variant genotypes and minor allele Del (Ins/Del + Del/Del) categorization. The *VAMP2* mRNA expression level were obtained from PBMCs of 435 subjects (139 CT and 296 cognitively impaired subjects with *VAMP2* 26 bp polymorphism genotyping available data) out of the 674 enrolled in the cohort. For the remaining 239 excluded subjects the PBMCs sample was exhausted in our repository or the *VAMP2* 26 bp polymorphism genotyping was missing.

**FIGURE 1:**
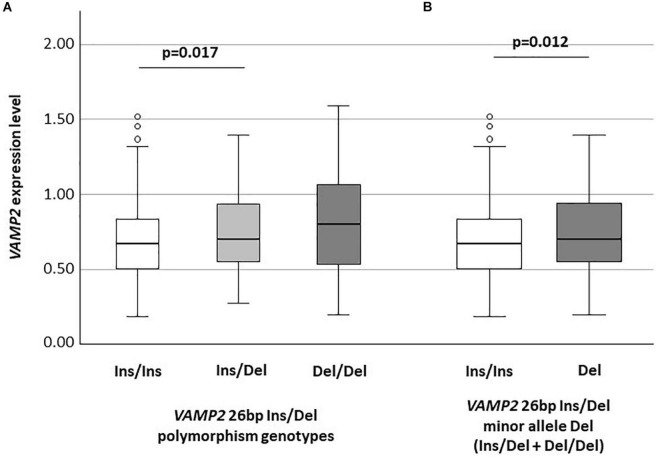
*VAMP2* mRNA expression according to *VAMP2* 26 bp Ins/Del polymorphism **(A)** and to *VAMP2* 26 bp minor allele Del (Ins/Del + Del/Del) **(B)**.

*VAMP2* mRNA expression was significantly different among the Ins/Ins (n: 314; median: 0.67: IQR: 0.50–0.84), Ins/Del (n: 109; 0.70: 0.55–0.93) and Del/Del (n: 12; 0.80: 0.53–1.07) genotypes (*p* = 0.043). In particular, *VAMP2* expression was significantly increased in Ins/Del compared to Ins/Ins genotype (*p* = 0.017) (Figure 1A); *VAMP2* mRNA expression was also consistently increased in Del/Del compared to Ins/Del and Ins/Ins carriers; these differences approached but did not reach statistical significance. *VAMP2* expression was evaluated next according to *VAMP2* minor allele Del categorization. Results showed the presence of a significant difference between Ins/Ins (n: 314; 0.67: 0.50–0.84) and minor allele Del (n: 121; 0.70: 0.55–0.94) (*p* = 0.012) (Figure 1B).

### *VAMP2* mRNA Expression Level According to Cognitive Impairment

The *VAMP2* mRNA expression level obtained from available PBMCs of 438 subjects out of the 674 enrolled were next evaluated according to cognitive impairment (73 AD, 122 MD, 140 CT and 103 MCI of which 18 MCI-MD and 19 MCI-AD). For the remaining 236 subjects (104 AD, 10 MD, 110 CT and 12 MCI of which 2 MCI-MD and 3 MCI-AD) the PBMCs sample was exhausted in our repository.

**FIGURE 2:**
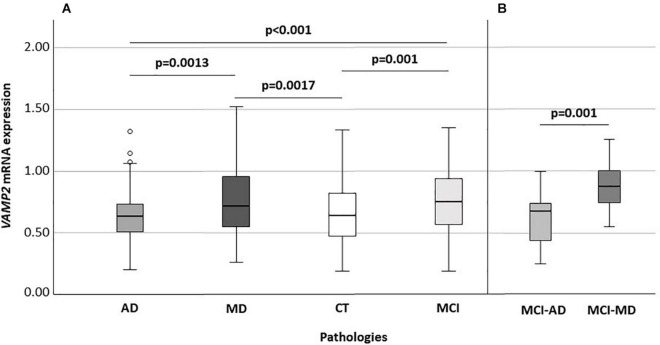
*VAMP2* expression according to diseases **(A)** and according to the MCI evolution **(B)**. Global significance: *p* < 0.001 **(A)**. AD, Alzheimer’s Disease; MD, mixed dementia; CT, subject without cognitive decline; MCI, Mild Cognitive Impairment; MCI-AD, MCI individuals evolved to AD; MCI-MD, MCI individuals evolved to mixed dementia.

*VAMP2* mRNA expression was significantly different when AD (n: 73; 0.63: 0.50–0.73), MD (n: 122; 0.71: 0.55–0.95), MCI (n: 103; 0.75: 0.56–0.94) and CT (n: 140; 0.63: 0.47–0.81) individuals were compared (*p* < 0.001) (Figure 2A). In particular, *VAMP2* expression was significantly increased in MCI compared to AD (*p* < 0.001) and CT (*p* = 0.001) and in MD compared to AD patients (*p* = 0.0013) and CT (*p* = 0.0017) (Figure 2). To note, no difference in *VAMP2* mRNA expression was found in CT compared to AD patients (*p* = 0.64), or between MCI and MD patients (*p* = 0.78).

Considering MCI subjects with known clinical evolution, at recruitment, when all these individuals had a diagnosis of MCI, *VAMP2* expression was significantly augmented in MCI that converted to MD (n: 18) compared to those converted to AD (n: 19) (0.87: 0.74–1.00 vs. 0.67: 0.42–0.74, *p* = 0.001) (Figure 2B). Binomial logistic regression analysis confirmed that *VAMP2* gene expression (*p*_*c*_ = 0.004) was significantly increased in MCI-MD compared to MCI-AD after correction for age, gender, MMSE and *ApoE4* positivity/negativity.

### Receiver Operating Characteristic Curve Analysis

**FIGURE 3:**
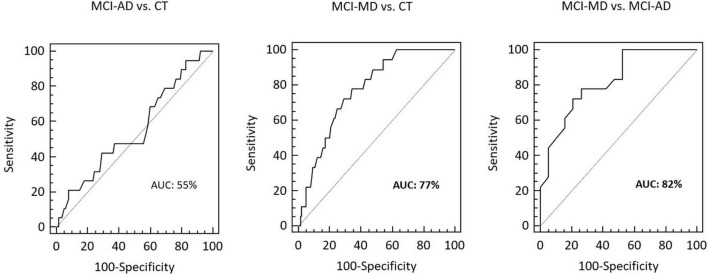
The ROCs curve of *VAMP2* gene expression for MCI-AD vs. CT, MCI-MD vs. CT and MCI-MD vs. MCI-AD evaluation. ROC, receiver operating characteristic; AUC, area under the curve.

Receiver operating characteristic curve analysis was employed to evaluate the ability of *VAMP2* gene expression to discriminate between clinically different forms of dementia. Eighteen MCI-MD, 19 MCI-AD and 140 CT subjects were considered in the analyses. The total area under the curve (AUC) of MCI-AD vs. CT was 55% (95% CI: 0.47–0.63) and of MCI-MD vs. CT was 77% (95% CI: 0.70–0.83; sensitivity: 77.8%, 95% CI: 52.4–93.6; specificity: 65.7%, 95% CI: 57.2–73.5) (Figure 3). Finally, the AUC of MCI-MD vs. MCI-AD was 82% (95% CI: 0.66–0.93; sensitivity: 77.8%, 95% CI: 52.4–93.6; specificity: 73.7%, 95% CI: 48.8–90.9) (Figure 3).

### *VAMP2* 26 bp Ins/Del Polymorphism Distribution in Alzheimer’s Disease, Mixed Forms of Dementia and Subject Without Cognitive Decline

Molecular genotyping of *VAMP2* 26 bp Ins/Del polymorphism was performed in all the subjects; distribution analysis was done grouping AD patients together with MCI subjects which developed AD (AD/MCI-AD) and MD patients together with MCI subjects that developed MD (MD/MCI-MD). The 73 MCI with unknown clinical evolution were excluded from this analysis.

*VAMP2* 26 bp Ins/Del polymorphism distribution was in Hardy–Weinberg equilibrium in all the groups (*p* > 0.05), but *VAMP2* 26 bp Ins/Del polymorphism genotypes distribution was significantly different among the three analyzed groups (*p* = 0.032; d.f.: 4; χ^2^ = 10.56). Thus, results showed that the Del/Del homozygote genotype was more frequently present in MD/MCI-MD (4.6%) compared to AD/MCI-AD and CT (1 and 0.8%, respectively) (Table 3). In particular, a significant different genotype distribution was seen in MD/MCI-MD compared to CT (*p*_*c*_ = 0.036; d.f.: 2; χ^2^ = 6.65) and a trend of significance in MD/MCI-MD compared to AD/MCI-AD (*p*_*c*_ = 0.096; d.f.: 2; χ^2^ = 4.67). Finally, although *VAMP2* 26 bp Ins/Del allelic distribution analysis did not show the presence of significant differences between the three analyzed groups (*p*_*c*_ = 0.13; d.f.: 2; χ^2^ = 4.06), the Del allele was more frequent in MD/MCI-MD compared to CT showing a trend of significance (*p* = 0.05; OR: 1.51; 95% CI: 0.99–2.28).

**TABLE 3:** Genotypes and allelic distribution of *VAMP2* 26 bp Ins/Del polymorphism in AD, MD patients and CT subjects.

*VAMP2* 26 bp Ins/Del (%)	Del/Del	Ins/Del	Ins/Ins	Del (Del/Del + Ins/Del)	Ins	Del
MD/MCI-MD^2^ (*n* = 152)	4.6	23.0	72.4	27.6	83.9	16.1^3^
AD/MCI-AD (*n* = 196)	1.0	26.5	72.5	27.5	85.7	14.3
CT^1^ (*n* = 248)	0.8	20.9	78.3	21.7	88.7	11.3

*AD, Alzheimer’s disease; CT, subject without cognitive decline; MD, mixed dementia; MCI-AD, Mild Cognitive Impairment evolved to Alzheimer’s Disease; MCI-MD, Mild Cognitive Impairment evolved to Mixed Dementia.*

*^1^AD/MCI-AD vs. MD/MCI-MD vs. CT: p = 0.032; d.f.: 4; χ^2^ = 10.56; ^2^MD/MCI-MD vs. CT: p = 0.036; d.f.: 2; χ^2^ = 6.65; ^3^MD/MCI-MD vs. CT: p = 0.05; OR: 1.51; 95% CI: 0.99–2.28.*

## Discussion

In the present paper we studied *VAMP2* gene expression and *VAMP2* 26 bp polymorphism distribution in a cohort of elderly persons with different forms of cognitive impairment and dementia. Results showed that *VAMP2* mRNA is more expressed in PBMCs from MD patients compared to AD; notably, higher levels of *VAMP2* expression seems to occur even in the first stages of disease and, in particular, in those MCI patients who converted to MD compared to those who converted to AD. These results were also reinforced by the ROC analysis, which confirmed the specific discriminatory power of *VAMP2* expression level between MCI-MD and MCI-AD.

Additionally, results indicated that *VAMP2* mRNA expression is influenced by *VAMP2* genotype. Thus, subjects carrying the *VAMP2* 26 bp minor allele Del (Ins/Del + Del/Del) were characterized by higher *VAMP2* mRNA expression compared to those carrying the Ins/Ins genotype. How the 26 bp Ins/Del variant could influence the *VAMP2* expression is still unknown. It is important to note that several studies showed that Ins/Del intergenic variants could lead to higher gene transcription by influencing the affinity of the transcription machinery for the transcription binding site (Chen et al., 2007; Xu et al., 2018; Wong et al., 2019). Interestingly, the genotype distribution showed that the *VAMP2* 26 bp Ins/Del polymorphism associates with MD and with MCI-MD. In particular, the frequency of individuals carrying the *VAMP2* 26 bp Del/Del genotype was significantly increased in MD patients compared to AD and CT subjects. This higher frequency of *VAMP2* 26 bp Del among the MD patients may account for the higher *VAMP2* mRNA expression seen in mixed form of dementia. Although being preliminary, these results strongly suggest that *VAMP2* mRNA expression is a possible early indicator able to distinguish MCI evolution to MD or AD as reinforced by the ROC evaluation and reinforce the hypothesis that *VAMP2* is a crucial gene in mixed form of dementia, but not in AD (Costa et al., 2019).

VAMP2 is a vesicle SNARE protein that plays a crucial role in neural communication and plasticity together with the target SNARE proteins STX1a and SNAP-25, through the formation of the SNARE complex. Impairments in SNARE complex assembly, changes in SNARE protein expression, and genetic variants of SNARE protein are widely observed in human as well as in mouse models of neurological diseases, and these alterations might be related to disease pathology and progression (Braida et al., 2015; Yang et al., 2015; Salpietro et al., 2019). Defects in this machinery can result in a range of neurological disorders associated to dementia (Costa et al., 2019; Melland et al., 2021). In particular, a reduction in the SNARE complex efficiency led to a lower neurotransmitters release inducing neurodegeneration (Haberman et al., 2012). In particular, a reduction in presynaptic proteins was found in the temporal cortex of VAD patients, even if this reduction was not nearly as important as what has been described in AD (Sinclair et al., 2015). Dysfunction in SNARE complex was also indicated as the initial trigger of accumulation of α-Synuclein (Nakata et al., 2012) in DLB, and a recent study showed the physical interaction of α-Synuclein with VAMP2 to promote SNARE assembly (Burré et al., 2010) in this condition. Finally, a reduction of Synaptophysin, a presynaptic protein that binds VAMP2 and plays a role in SNARE complex assembly and vesicle fusion, was observed to be present in FTD patients (Valtorta et al., 2004), suggesting a fundamental role of synaptic decline in dementia (Clare et al., 2010). *VAMP2* involvement, both at mRNA and protein levels, in the pathogenesis of VAD and in different forms of well-defined dementia, indeed suggest a possible role for this gene in the vascular alteration seen in the mixed form of dementia, that will need to be further investigated. Moreover, our data need further studies confirming levels of VAMP2 in central nervous system (brain tissue or liquor). Anyway, PBMCs may serve as a peripheral laboratory to find molecular signatures that could aid in differential diagnosis with other forms of dementia and in monitoring of disease progression (Arosio et al., 2014). The number of patients we analyzed is relatively small, in particular regarding the MCI subjects with a clinically defined evolution, and as a consequence these findings will need to be confirmed in larger cohorts of patients. Longitudinal studies and analyses focusing on the impact of the 26 bp Ins/Del variant on *VAMP2* gene and protein expression will also shed more light on the role of VAMP2 in the pathogenesis of dementia.

These caveats notwithstanding, the identification of easy-to-collect biomarker such as *VAMP2* able to discriminate between different forms of mixed form of dementia could be useful to allow early therapeutic and/or rehabilitative intervention, with beneficial efforts for the patients, caregivers and the public health system.

## Data Availability Statement

The original contributions presented in the study are included in the article/supplementary material, further inquiries can be directed to the corresponding author/s.

## Ethics Statement

The studies involving human participants were reviewed and approved by Ethical Committee of the IRCCS Fondazione Ca’ Granda Ospedale Maggiore Policlinico (protocol number: 00223248). The patients/participants provided their written informed consent to participate in this study.

## Author Contributions

AC, FG, and BA conceived and designed the project. AC, EF, and PR assisted with biological samples and data collection. AC and EF performed the experiments and data analysis. MC supervised the project. AC and FG wrote the manuscript. All authors read and approved the final manuscript.

## Conflict of Interest

The authors declare that the research was conducted in the absence of any commercial or financial relationships that could be construed as a potential conflict of interest.

## Publisher’s Note

All claims expressed in this article are solely those of the authors and do not necessarily represent those of their affiliated organizations, or those of the publisher, the editors and the reviewers. Any product that may be evaluated in this article, or claim that may be made by its manufacturer, is not guaranteed or endorsed by the publisher.
